# Plant-derived Ren's oligopeptide has antiviral effects on influenza virus and SARS-CoV-2

**DOI:** 10.3389/fvets.2022.1090372

**Published:** 2023-02-02

**Authors:** Chengzhi Yu, Yayu Huang, Xin Ren, Leqiang Sun

**Affiliations:** ^1^College of Veterinary Medicine, Huazhong Agricultural University, Wuhan, China; ^2^Wuhan Shiji Maide Biotechnology Company, Wuhan, China; ^3^State Key Laboratory of Agricultural Microbiology, Huazhong Agricultural University, Wuhan, China

**Keywords:** influenza virus, SARS-CoV-2, Ren's oligopeptide, antiviral drugs, virus replication

## Abstract

Influenza virus and SARS-CoV-2 virus are two important viruses that cause respiratory tract diseases. The high-frequency mutation of the two types of viruses leads to failure of the durable immune protection of vaccines, meanwhile it also poses continuous challenges to the development of antiviral drugs. Traditional Chinese medicine contains large number of biologically active compounds, and some of them contain broad-spectrum antiviral ingredients. In this study, we extracted antiviral active ingredients from medicinal and edible plants by biotransformation and enzymatic hydrolysis as a drug, and we named this drug Ren's oligopeptide. Further, we analyzed the antiviral activity of this drug and found that Ren's oligopeptide could inhibit the replication of influenza virus and SARS-CoV-2 virus with high anti-virus activities. *In vitro* experiments showed that the antiviral activity of the Ren's oligopeptide mainly targets the replication process after virus enters the cell. Therefore, Ren's oligopeptide is a promising drug against influenza and COVID-19.

## Introduction

Influenza is highly contagious, which cause huge economic losses. Therefore, influenza is listed as one of the major infectious diseases that seriously threaten human health (1). Currently, vaccination and antiviral drug development are effective methods to fight against the influenza virus. However, influenza vaccine antigens need to be constantly updated to cope with the virus mutation caused by frequent recombination of viral gene segments (2). The drugs used for treating influenza virus infection mainly include oseltamivir, rapamycin, and peramivir (3–5). However, the side effects of the drug cannot be ignored, such as diarrhea and dizziness (6, 7). With the widespread use of drugs and the high variability of influenza viruses, the emergence of virus resistance to drugs becomes a great concern (8, 9).

The new coronavirus pneumonia caused by SARS-CoV-2 virus poses a huge challenge to global public health, causing 560 million infections and 8.67 million deaths worldwide by July 2022 (10). Currently, urgently-approved clinical drugs such as molnupiravir, remdesivir, favipiravir, lopinavir, and ritonavir are used for COVID-19 treatment (11–15). Unfortunately, the specificity and clinical therapeutic effect of these drugs are not desirable (16, 17). The mutation of the virus and the aggravation of the epidemic have entered a vicious circle, which poses difficulties to the global fight against the epidemic (16, 17).

Influenza virus and SARS-CoV-2 virus are two important pathogen causing respiratory tract infection, both viruses are highly contagious with high rates of mutation, which makes clinical antiviral treatment difficult (18). Chinese herbal extracts have direct or indirect antiviral effects on different periods of viral infection by targeting key steps in the viral replication cycle and key cellular components of the host defense system (18, 19). For example, pentagalloyl glucose extracted from the plant emblica can inhibit the release of virus particles (20), and dendrobine extracted from dendrobium interferes with early virus replication (21). In addition, many plant extracts can also exert an indirect antiviral effect by regulating the host immune function (22, 23). For example, quercetin and apigenin have antioxidant and anti-inflammatory properties, which can improve immunity of host cells (24). Some plant extracts have broad-spectrum antiviral properties. Allicin in garlic is a natural broad-spectrum antibiotic, which has certain inhibitory activity against herpes simplex virus, vesicular stomatitis virus, human rhinovirus, and influenza virus (25). Chinese herbal extracts provide abundant resources for developing broad-spectrum antiviral drugs, such as SARS-CoV-2 and influenza viruses (26–28). In this study, we report a new drug fermented from multiple edible plants, and this drug exhibits high inhibitory activity against the influenza A and SARS-CoV-2 virus *in vitro*. Therefore, it can be used as a candidate drug for the treatment of respiratory tract infections caused by influenza A virus and SARS-CoV-2 virus.

## Materials and methods

### Drug preparation

Ren's oligopeptide is prepared from six plant ingredients through enzymatic fermentation. Specifically, the fermented raw materials were chopped and mixed at the proportions of 20% ginger, 20% garlic, 20% onion, 10% banana peel, 20% bitter melon, and 10% deionized water. The fermentation enzyme (Amylase enzyme) was extracted from the Geotrichum candidum strain, and the concentration was measured by the QuantiPro ™ BCA kit (NA.32, Sigma, US). Amylase enzyme was added to the mixture at a weight ratio of 1:10,000, followed by 8-day aseptic fermentation at 25°C and the precipitate removal. The concentration of flavonoid anthocyanin in the products was determined to measure the degree of fermentation. Afterwards, the supernatant was sterilized and stored in aliquots at 4°C. The color of Ren's oligopeptide solution is brown and the concentration of Ren's oligopeptide was 900 μg/ml.

### Cell lines and virus

MDCK (ATCC CCL-34) and Vero-E6 (ATCC CCL-81) cells were passaged in DMEM medium (Gibco, Dulbecco's Modified Eagle Medium, 11965092, Thermo Fisher, USA) containing 10% FBS (Gibco, Fetal Bovine Serum, 10099141C, Thermo Fisher, USA) and 1% penicillin-streptomycin (Gibco, 10378016, Thermo Fisher, USA). When reached 95% confluency, the cells were digested with 0.25% trypsin (Gibco, 25200072, Thermo Fisher, USA) and transferred into flasks, then incubation at 37°C and 5% CO_2_ for 48 h. The influenza and SARS-CoV-2 virus strain used in this study were H1N1-PR8 and nCOV2019BetaCoV/Wuhan/WIV04/2019.

### Cytotoxic assays

MDCK and Vero-E6 cells were separately seeded into 96-well plates at 1 × 10^4^ cells per well and incubated at 37°C and 5% CO_2_ for 24 h. Ren's oligopeptide was two-fold serially diluted in PBS, then added to the 96-well plate with 6 replicates per group. In addition, a drug-free group treated with PBS was used as control group. Cell viability was detected with Cell Counting Kit 8 (Abcam, ab228554, UK) after 48 h incubation. The 10 μl/well of WST-8 solution was added into each well, and then incubate protected from dark at 37°C for 2 h. The absorbance at 450 nm was measured by a microplate reader.

### Antivirus assays

To investigate the antiviral effect of Ren's oligopeptide, TCID50 was performed. The experiment grouping was as follows: (1) control group: cells without virus infection; (2) virus infection group: cells were incubated with 100 TCID50 viruses; (3) virus pretreatment group: virus and Ren's oligopeptide were mixed at room temperature for 30 min before infection; (4) cell pretreatment group: cells were incubated with Ren's oligopeptide for 24 h and then infection with 100 TCID50 virus; (5) virus adsorption-blocking group: cells were treated with Ren's oligopeptide for 1 h during the virus adsorption period; (6) virus replication-blocking group: the diluted Ren's oligopeptide was added after virus adsorption.

The antiviral effect of Ren's oligopeptide was analyzed at its maximum non-toxic concentration. 1 × 10^4^ of MDCK or Vero-E6 cells per well were seeded into a 96-well plate, and cultured at 37°C and 5% CO_2_ for 24 h. The diluted drug (1:16) was added to the pretreatment group. An equal volume of PBS was added to other groups. After 24 h incubation, each group was infected with the virus with a dose of 100 TCID50. The supernatants were collected after 48 h incubation for RNA extraction, and the virus copy number was detected by qRT-PCR.

To test the dose-dependent antiviral effect of Ren's oligopeptide, the drug was two-fold serially diluted with its maximum non-toxic concentration as initial concentration. After infection for 1 h, the diluted drug was added to the virus replication-blocking group. After 48 h cell culture, the supernatant was collected for RNA extraction. The experiments were performed in triplicates.

### Quantitation of virus copy number

For SARS-CoV-2 virus, the cell supernatants were centrifuged at 3,000 rpm for 5 min, then virus lysate was added, followed by virus inactivation according to Wuhan National Biosafety Laboratory BSL-3 SOP. Viral RNA was extracted with Takara viral RNA extraction kit (Takara, 9,767, Japan), and the viral copy number was detected by qRT-PCR using the Luna Universal One-Step RT-qPCR Kit (New England Biolabs, E3005S, USA). Plasmid pUC57-RBD contains receptor-binding domain (RBD) sequence was used to generate a standard curve. The primers pair for SARS-CoV-2 detection were RBD-F (CAATGGTTTAACAGGCACAGG) and RBD-R (CTCAAGTGTCTGTGGATCACG), and the detection sequence is 5'6FAM-ACAGCATCAGTAGTGTCAGCAATGTCTC-3'BHQ1. The H1N1 influenza viral genomic RNA was extracted using a nucleic acid extractor, and the viral copy number was detected by qRT-PCR (New England Biolabs, E3005S, USA). Standard curves were created using plasmid pUC57-NP harboring the nucleoprotein (NP) sequence. The primers pair for H1N1 virus detection were NP-qF (GAGCCGGAACCCAGGGAATGCTGA) and NP-qR (CAAAGTCGTACCCACTGGCTACGGCAG), and the detection sequence is 5'6FAM-TCCATACACACAGGCAGGCAGGCAGGAC-3'BHQ1. All the experiments were performed in triplicates.

### Statistical analysis

The virus inhibition rate was calculated according to the following formula:

Virus inhibition rate = (1-viral copy number in drug treatment group/ viral copy number in virus infection group) × 100%. CC50 (50% cytotoxicity concentration) and EC50 (50% effective inhibition concentration) were calculated by non-linear regression according to cell viability and viral inhibition rate. The *t*-test was conducted to assess the statistical significance. *P* < 0.05 was considered statistically significant.

## Results

### Cytotoxicity assay

**Figure 1:**
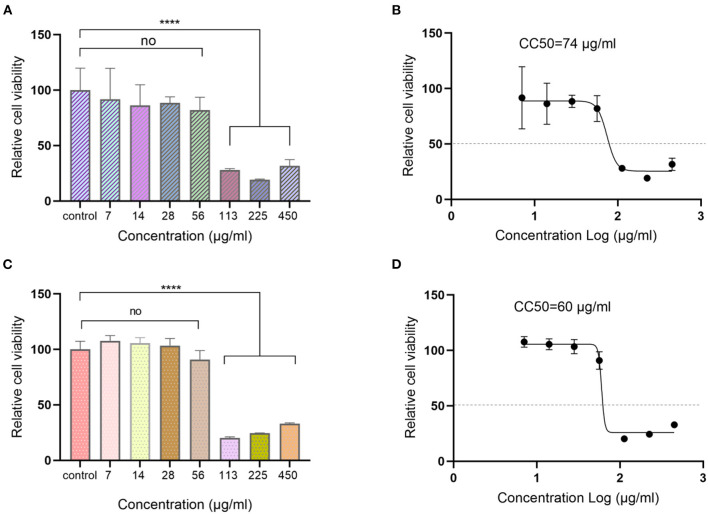
Cytotoxicity assay of Ren's oligopeptide. **(A)** Viability of MDCK cells at different concentrations of Ren's oligopeptide. **(B)** 50% cytotoxicity concentration (CC50) of Ren's oligopeptide on MDCK cells. **(C)** Viability of Vero-E6 cells at different concentrations of Ren's oligopeptide. **(D)** 50% cytotoxicity concentration (CC50) of Ren's oligopeptide on Vero-E6 cells. ns, not significant (*p* > 0.05); ****p < 0.0001 from the Student's *t*-test. Error bars represent SD from three independent experiments.

MDCK and Vero-E6 are the commonly used cell lines for influenza virus and SARS-CoV-2 virus infection. The cytotoxic effects of Ren's oligopeptide on the above-mentioned two cell lines were analyzed before antiviral efficiency evaluation to determine the maximum drug non-toxic concentration. Cells were incubated with different concentrations of the Ren's oligopeptide for 48 h, and the cell viability was assessed by CCK8 kit. We observed that cells grew well in the 56 μg/ml treatment group and the viability of MDCK cells was 85.42%, which was not significantly different from that in the control group (*P* = 0.34). However, the cell viability was significantly decreased in the 113 μg/ml treatment group (28.29%, *P* < 0.0001) (Figure 1A). Similarly, when the drug concentration was 56 μg/ml, no significant difference in the viability of Vero-E6 cells was observed between drug treatment group and the control group (90.94%, *P* = 0.64), and the cell viability was significantly lower in drug treatment group than in the control group when the drug concentration was 113 μg/ml, (<20.33%) (Figure 1C). Finally, the CC50 of the drug was determined as 74 μg/ml for MDCK cells and 60 μg/ml for Vero-E6 cells (Figures 1 B, D). Since the maximum non-toxic concentration was achieved at 56 and 56 μg/ml was determined as the optimal concentration for the subsequent antiviral analysis.

### Anti-influenza virus activity

**Figure 2:**
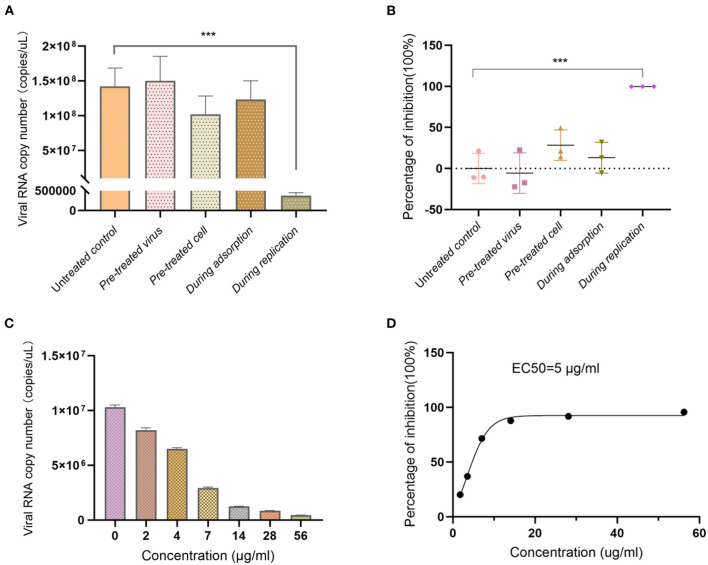
Anti-influenza virus effect of Ren's oligopeptide. **(A)** Virus copy number of H1N1 after addition of Ren's oligopeptides to the MDCK cells at different viral infection stages. **(B)** Virus inhibition rate after addition of Ren's oligopeptide to cells at different viral infection stages. **(C)** Inhibition rate at different concentrations of Ren's oligopeptides on H1N1 virus. **(D)** Ren's oligopeptide anti-H1N1 dose-dependent curve. ns, not significant (*p* > 0.05); ***p < 0.001 from the Student's *t*-test. Error bars represent SD from three independent experiments.

In order to investigate the antiviral effect of Ren's oligopeptide, the drug at the maximum non-toxic concentration was added to the cells in different virus infection stages. The results showed that the replication of the virus was not inhibited in the virus pretreatment group (*P* = 0.71), indicating that the drug had no direct killing effect on the virions. Compared with that in the control group, the virus copy number in the cell pretreatment group was decreased with an inhibition rate of 28.34%, but there was no significant difference (*P* = 0.085). The inhibition rate in the virus adsorption blocking group was 13.19% with no significant difference from the control group (*P* = 0.394). When the drug was added to cells during viral replication, the replication of the virus was significantly reduced with an inhibition rate of 99.74% (*P* = 5.1 × 10^−5^) (Figures 2 A, B). Overall, these results showed that Ren's oligopeptide had a significant inhibitory effect on the replication of the influenza virus (H1N1) by targeting the replication stage of the virus.

Further, we analyzed whether the antiviral effect of Ren's oligopeptide was dose-dependent by serial dilution. Our results showed that with the decreasing drug concentration, the virus copy number was increased (Figure 2C), and the virus inhibition rate decreased. When the concentration ranged from 14 to 56 μg/ml, the drug exhibited a strong inhibitory effect on virus replication with an inhibition rate above 85% (Figure 2C). In the 2 μg/ml treatment group, the virus inhibition rate was 20%. The results showed that the anti-H1N1 effect of Ren's oligopeptide was dose-dependent with an EC50 of 50.4 μg /ml (Figure 2D).

### Anti-SARS-CoV-2 virus activity

**Figure 3:**
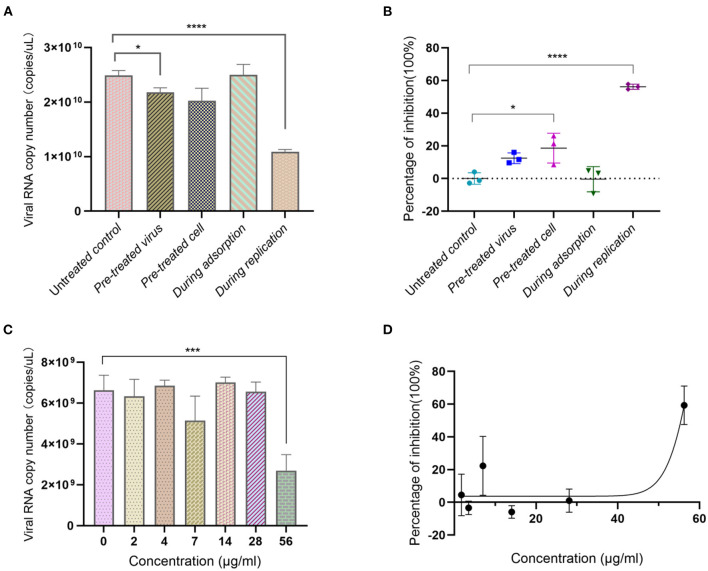
Anti-SARS-CoV-2 virus effect of Ren's oligopeptide. **(A)** Virus copy number of SARS-CoV-2 after addition of Ren's oligopeptides to the Vero-E6 cells at different virus infection stages. **(B)** Inhibition rate of SARS-CoV-2 virus after adding Ren's oligopeptides to the Vero-E6 cells at different virus infection stages. **(C)** Inhibitory rate of Ren's oligopeptides at different concentrations on SARS-CoV-2. **(D)** Ren's oligopeptide anti-SARS-CoV-2 dose-dependent curve. ns, not significant (*p* > 0.05); *p < 0.05, ***p < 0.001, and ****p < 0.0001 from the Student's *t*-test. Error bars represent SD from three independent experiments.

We assessed the anti-SARS-CoV-2 virus effect of Ren's oligopeptide against SARS-CoV-2 using Vero-E6 cells. The results showed that the virus copy number in virus pretreatment group displayed a downward trend with an inhibition rate of 12.45% (*P* = 0.025). The virus copy number was 7.27% lower in the cell pretreatment group than in the control group, but it was not statistically significant (*P* > 0.99). When it was added to the Vero-E6 cells during the virus adsorption stage, the drug did not show any inhibitory effect. However, when the drug was added 1 h after virus infection, the virus replication was significantly lower than that in the control group (*P* = 1.56 × 10^−5^) with an inhibition rate of 56.34% (Figures 3A, B). Similarly, we determined whether the anti-SARS-CoV-2 activity of Ren's oligopeptide was dose-dependent. In the 56 μg/ml treatment group, the drug showed a significant anti-virus effect with a virus inhibition rate of 59.29%. However, at other dilution ratios, the drug did not show a significant virus-inhibitory effect (Figures 3C, D). Therefore, the anti-SARS-CoV-2 effect of Ren's oligopeptides was not dose-dependent.

## Discussion

Ren's oligopeptide is fermented from edible plant ingredients with a wide range of raw materials and low side effects. In this study, we confirmed Ren's oligopeptide antiviral activity against SARS-CoV-2 virus and influenza virus *in vitro*. Ren's oligopeptide showed a significant antiviral effect on the influenza virus H1N1 at the maximum non-toxic concentration with an anti-H1N1 inhibition rate of up to 99%. The direct incubation of the drug with the virus did not inhibit the replication of the H1N1 virus, indicating that the drug could not directly kill the virus to exert its antiviral effect. The addition of the drug during virus adsorption stage only resulted in a limited antiviral effect. In both the H1N1 infection group and the SARS-CoV-2 infection group the addition of drug during the virus replication stage exhibited the optimal inhibitory effect on the virus replication, indicating that the drug mainly played a role by targeting the virus replication stage.

The antiviral effect of Ren's oligopeptides results from the synergistic action of various active ingredients in the fermentation materials. Garlic is known as a natural antibiotic with broad-spectrum antiviral effects (25). Organosulfurs (such as allicin and alliin) and flavonoids (such as quercetin) are the main antiviral components of garlic extract (29). The antiviral potential of garlic has been widely reported against various viruses such as HIV (30), dengue virus (31), herpes simplex virus (32), and influenza virus (33). The anti-influenza activity of garlic extract may be due to inhibition of viral replication or blocking virus budding from infected cells (33). A computer virtual screening study showed that the organosulfur and flavonoids of garlic can inhibit SARS-CoV-2 virus replication through the interaction with Mpro protease (34). Onions contain allicin, quercetin, and other sulfur-containing compounds (35). Onion extract has natural antiviral and immune enhancement effects, and onion extract can reduce the mortality of avian infected with Newcastle disease virus by preventing the virus from attaching to the cell (36). Increasingly studies have shown that gingerol and shogaol in ginger have various biological activities, including antioxidant, anti-inflammatory, antibacterial, and other activities (37). Gingerenone A, a ginger extract, restricts influenza virus replication by inhibiting JAK2 activity (38). The ribosome-inactivating proteins (RIPs) in bitter melon have antiviral activity (39), and a type of plant protein extracted from bitter melon exhibits antiviral activity against influenza A virus (40). The above plant components contribute to the direct antiviral activity of Ren's oligopeptide. Ren's oligopeptide contains a variety of antiviral components from different sources, and thus Ren's oligopeptide has broad-spectrum antiviral activity, and it is capable of inhibiting both H1N1 and SARS-CoV-2 replication. In addition to direct antiviral activity, garlic, onion, ginger, and banana peels exhibit an indirect antiviral effect. For example, alliin in garlic has immune-enhancing effects, which can mediate the innate immune response by macrophages and NK cells (41). Ginger and its active compounds can effectively alleviate the inflammatory response by inhibiting the activation of NF-κB and enhancing the expression of anti-inflammatory cytokines (42). The high content of phenolic and flavonoid compounds in banana peels have antioxidant and radical-scavenging abilities (43). The above components can enhance antiviral effect by improving the immunity of host cells. Furthermore, garlic, ginger, and onion extracts have been reported to be used in clinical pneumonia rehabilitation by relieving the abnormal alveolar space, alveolar wall thickening, lung cell infiltration and pulmonary fibrosis symptoms, and these extracts contribute to the recovery from respiratory virus infection symptoms (44).

Based on the above findings, we proposed that Ren's oligopeptide can be used as a candidate drug for the treatment of respiratory diseases caused by influenza virus and SARS-CoV-2 virus infection. The limitation of this study is that the detail antiviral mechanism and *in vivo* antiviral effect of this drug remain to be further investigated.

## Conclusions

Ren's oligopeptide is fermented from edible plants with antiviral effects, it contains a variety of antiviral active compounds. Through the *in vitro* antiviral analysis, we determined that the maximum non-toxic concentration of Ren's oligopeptide is 56 μg/ml, and Ren's oligopeptide has a significant antiviral effect on influenza virus and SARS-CoV-2 virus, and the antiviral activity is mainly manifested in the replication phase after the virus enters the cell.

## Data availability statement

The original contributions presented in the study are included in the article/supplementary material, further inquiries can be directed to the corresponding author.

## Author contributions

CY: conceptualization. CY and YH: methodology and formal analysis. XR: software and investigation. YH and XR: validation. LS: resources and writing and editing. LS and CY: data curation and project administration. All authors contributed to the article and approved the submitted version.
